# Chikungunya fever: a re-emerging mosquito-borne threat demanding proactive prevention strategies in Asia

**DOI:** 10.3389/fpubh.2026.1745145

**Published:** 2026-01-23

**Authors:** Sophie So Wan Yip, Caroline Yee Chong Charm, Kei Shing Ng, Simon Ching Lam

**Affiliations:** 1 School of Nursing and Health Sciences, Hong Kong Metropolitan University, Hong Kong, Hong Kong SAR, China; 2 Department of Diagnostic Radiology, Li Ka Shing Faculty of Medicine, The University of Hong Kong, Pok Fu Lam, Hong Kong SAR, China; 3 School of Nursing, Tung Wah College, Ho Man Tin, Hong Kong SAR, China

**Keywords:** chikungunya fever, aedes, arboviruses, public health surveillance, vector control

## Introduction

Chikungunya fever is an acute mosquito-borne viral disease caused by the chikungunya virus (CHIKV), an alphavirus primarily transmitted by *Aedes aegypti* and *Aedes albopictus* (1). It was first identified in Tanzania, Africa, in the 1950s, and historically remained largely confined to parts of Africa and Asia. However, the virus has resurged globally in the past two decades, undergoing a remarkable geographic expansion with explosive outbreaks first noted in the Indian Ocean islands and subsequently sweeping through Asia and the Americas (2). This re-emergence established the virus in vast new populations, transforming it from a localized pathogen into a global public health threat. Characterized by the abrupt onset of high fever, severe arthralgia (joint pain), rash, and often debilitating chronic arthritis, chikungunya is seldom fatal but causes considerable morbidity and socioeconomic disruption, particularly in densely populated urban settings (3, 4).

As of late 2024, the autochthonous (local) transmission of CHIKV has been documented in over 110 countries and territories across the globe, establishing it as a major global health problem (5). The virus is endemic across wide swathes of Africa, Southeast Asia, the Indian subcontinent, the Pacific Region, and the Americas (5, 6). This extensive geographical spread is driven by the presence of the competent vectors *A. aegypti* and *A. albopictus*, coupled with factors, like increased global travel and climate change, which expands mosquito habitats (6). While historically confined to the tropics, the movement of infected travelers has led to small, localized outbreaks in temperate regions, such as those reported in Italy (2007) and France (2014), highlighting the persistent risk of importation and local establishment outside traditionally endemic zones (7). The Asian and East–Central–South African lineages are the genotypes predominantly circulating worldwide (6).

The diagnosis of CHIKV infection remains challenging, particularly in cocirculation settings where the virus circulates alongside dengue (DENV) and zika (ZIKV) (8). Although the initial clinical presentations of all three arboviruses substantially overlap, a distinguishing feature of CHIKV is the prominence of severe, symmetrical polyarthralgia and arthritis, which can persist for months to years, a characteristic less common in DENV or ZIKV infections (9). For definitive laboratory confirmation, reverse transcription polymerase chain reaction is the primary method for detecting viral RNA in the acute phase (within the first 5–7 days of illness) (10). After this window, serological assays (IgM/IgG ELISA) are employed, although the potential for cross-reactivity with other circulating flaviviruses necessitates further confirmation by specific, albeit resource-intensive, techniques, like the plaque reduction neutralization test (10). Currently, no specific antiviral treatment for chikungunya fever exists; management remains supportive, focusing on pain relief through analgesics and nonsteroidal anti-inflammatory drugs (NSAIDs). However, NSAIDs must be used cautiously and often excluded until DENV coinfection is ruled out (11). Prevention has been revolutionized by the recent introduction of the world's first licensed chikungunya vaccine, IXCHIQ^^®^^, with access rapidly expanding in endemic regions through agreements, such as the technology transfer and licensing agreements with the Serum Institute of India, aimed at ensuring priority supply in Asia (12).

Recent events in mainland China underscore the urgency of re-evaluating chikungunya as a regional public health concern. As of early December 2025, the Guangdong Province outbreak has escalated, resulting in over 16,000 confirmed cases, although the rate of new infections has been decreasing. The virus was likely imported via infected travelers and rapidly amplified through local *A. albopictus* populations, prompting a level 3 emergency response, which indicates that it is a major crisis and requires massive, organization-wide mobilization. These concerns were realized with Hong Kong recording its first locally acquired case of chikungunya fever this year on October 25, 2025, involving an 82-year-old woman in the Wong Tai Sin area. This situation has raised anxieties about the potential for local transmission, especially given Hong Kong's densely populated urban environment and the ongoing outbreak in nearby Guangdong Province, China.

This article presents a viewpoint on the importance of the 2025 China outbreak as a wake-up call for regional governments, particularly Hong Kong, to adopt proactive, evidence-based strategies for chikungunya prevention. By discussing the causes of transmission, gaps in vaccination and mosquito control, and the critical role of public health education, this paper aims to encourage timely surveillance, regional coordination, and targeted vector management efforts before outbreaks become endemic.

## Cause and transmission of CHIKV

**Figure 1:**
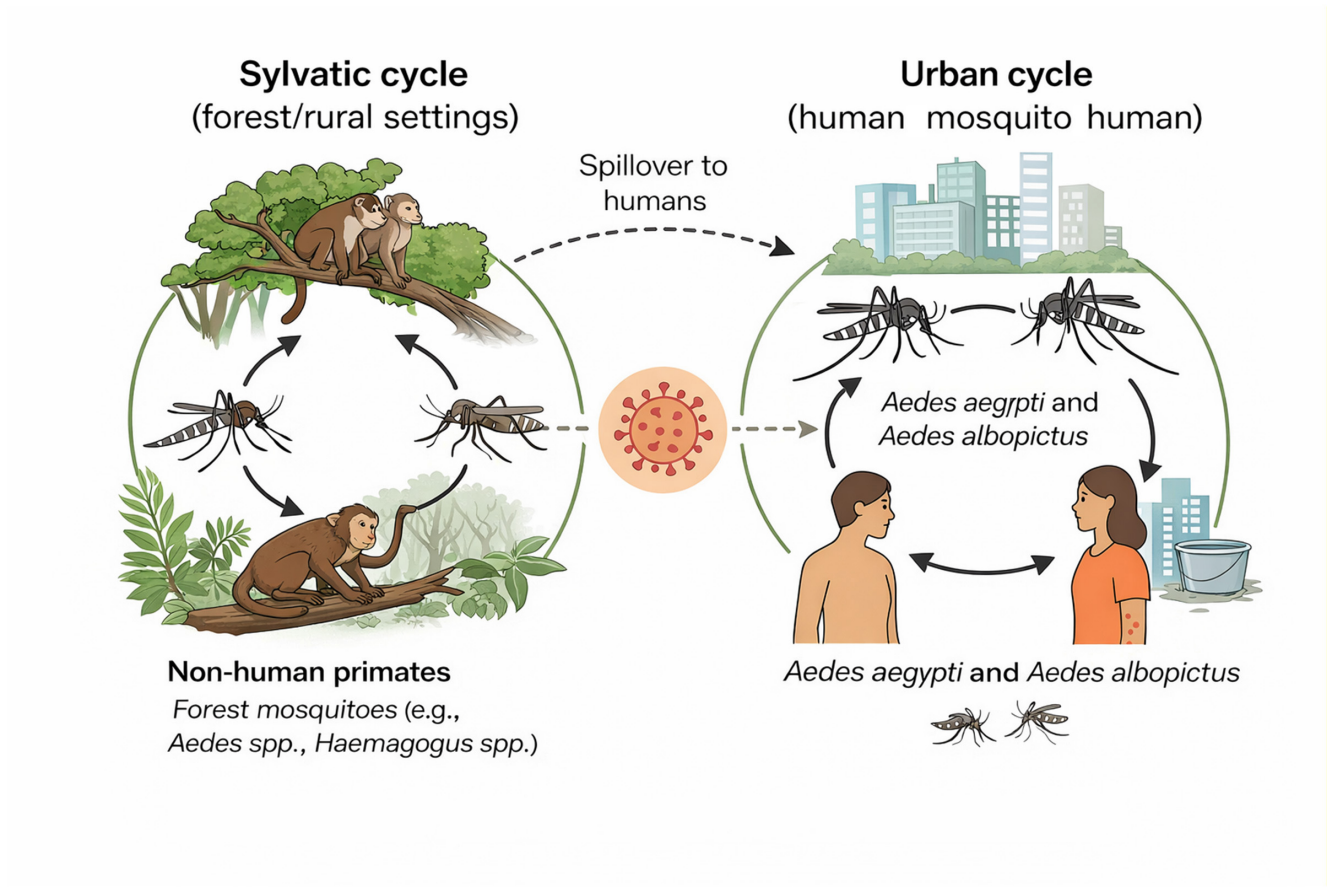
Transmission cycles of chikungunya virus (CHIKV). CHIKV is maintained through two distinct ecological pathways: the sylvatic (enzootic) cycle and the urban (epidemic) cycle. In the sylvatic cycle, the virus circulates between non-human primate reservoirs and forest-dwelling mosquito vectors (e.g., *Aedes furcifer, Ae. africanus*). Zoonotic spillover occurs when humans are occasionally infected through forest-related activities, potentially introducing the virus into urban settings. The urban transmission cycle involves a bidirectional exchange between human hosts and urban mosquito vectors, primarily *Aedes aegypti* and *Aedes albopictus*, facilitating rapid spread in densely populated areas [illustration created by the authors using Adobe Illustrator and adapted from “Transmission of Chikungunya Virus” (Centers for Disease Control and Prevention) available at: https://www.cdc.gov/chikungunya/php/transmission/index.html].

CHIKV is a single-stranded RNA virus of the genus *Alphavirus* and family Togaviridae (13). The virus maintains two primary, ecologically distinct transmission cycles: the sylvatic and urban cycles (13). The sylvatic cycle is the ancestral enzootic mechanism in Africa, where the virus is maintained between a non-human primate reservoir (such as monkeys and baboons) and forest-dwelling *Aedes* mosquitoes, including species like *Aedes africanus* and *Aedes furcifer*. Human infection in this cycle is typically an accidental spillover event, occurring when people encroach on forest habitats and are bitten by these bridge vectors (13). By contrast, the urban cycle is responsible for the massive outbreaks seen globally and operates as a human–mosquito–human transmission loop. This cycle is driven primarily by the highly domesticated vectors *A. aegypti* and *A. albopictus*. These mosquitoes are day biting, thrive in urban environments, and breed in stagnant water. *A. albopictus*, which is widely distributed across subtropical Asia, has been particularly instrumental in recent CHIKV outbreaks because of its adaptability to cool climates and urban–rural habitats (14), with viral adaptation (such as the A226V mutation) further enhancing its transmission competence (refer to Figure 1 for the transmission cycle of CHIKV). Beyond the main vector-borne (horizontal) route, non-vectorial modes of transmission include vertical transmission (mother to child), which is most dangerous when the mother is viremic during the perinatal period, as well as rare cases of blood-borne transmission via transfusions and occupational exposure in laboratory settings (15). Furthermore, while its public health importance has not been fully elucidated, CHIKV RNA has also been detected in semen and cervical samples, suggesting potential for sexual transmission (15).

Infected individuals typically develop symptoms within 3–7 days of being bitten. The clinical presentation includes abrupt fever onset, severe joint pain, muscle pain, rash, and fatigue (16). While mortality is rare, the disease can result in debilitating polyarthritis lasting weeks to months. Asymptomatic infections also occur, complicating case detection and increasing the risk of unnoticed transmission (17).

The potential for rapid transmission is compounded in regions with dense populations, insufficient vector control, and lack of community awareness. Seasonal rainfall and rising temperatures due to climate change further increase vector density and survival, making previously non-endemic regions increasingly vulnerable (18).

## Virus replication, viral kinetics, and immunopathogenesis

CHIKV is an enveloped virus with a positive-sense, single-stranded RNA genome (+ssRNA) that functions directly as a messenger RNA upon entry. The replication cycle begins when the viral E2 glycoprotein binds to cellular receptors, such as MXRA8, facilitating clathrin-mediated endocytosis. Once inside the endosome, low pH triggers a conformational change in the E1 glycoprotein, leading to fusion with the endosomal membrane and the release of genomic RNA into the cytoplasm (19). This genomic RNA is first translated into a non-structural polyprotein (P1234), which is subsequently cleaved by the viral protease nsP2 into four individual non-structural proteins (nsP1–nsP4) (20). These proteins assemble on modified intracellular membranes to form the replication complex, which uses the genomic RNA to synthesize a negative-sense RNA intermediate that then serves as the template for creating a new full-length genomic RNA and short subgenomic RNA (21). This subgenomic RNA encodes the structural capsid, E3, E2, 6K, and E1 proteins (22). The capsid protein then encapsidates the new genomic RNA to form the nucleocapsid, which subsequently buds through the plasma membrane, acquiring the envelope (containing the E1/E2 heterodimers) before being released as mature virions (23).

From an epidemiological perspective, the highest concentration of infectious virus and viral RNA (viremia) occurs during the acute phase of infection (Days 0–7 postsymptom onset), which is characterized by explosive viral replication in primary target cells, like fibroblasts and macrophages, making this window crucial for molecular diagnosis (PCR). Post Day 8, viral load typically declines as the adaptive immune response matures (24).

The immunology of CHIKV infection involves a robust response, including the early induction of type I interferons and the later development of neutralizing antibodies and activated CD4+ and CD8+ T cells that are essential for viral clearance from circulation and muscle tissue (25). However, the virus utilizes an immune-driven mechanism for chronic disease: while the adaptive system successfully clears the acute infection, it often fails to eliminate viral RNA and antigens from specific immune-privileged sites, particularly joint-associated tissues (e.g., synovial fibroblasts and macrophages). This persistent, low-level viral presence, often alongside persistent immune cell infiltration, acts as a continuous trigger for inflammation (synovitis and arthritis), effectively utilizing the host's own prolonged, dysfunctional adaptive immune response to drive the debilitating chronic polyarthralgia that can last months to years (26, 27).

## Recent outbreak dynamics and regional risks

The 2025 outbreak in Guangdong Province is a watershed moment for CHIKV epidemiology in China. What began as isolated imported cases quickly evolved into a large-scale outbreak, particularly concentrated in Foshan and its Shunde District, with thousands of cases confirmed in just weeks. Although regional health authorities responded with emergency surveillance, mosquito control measures, and community engagement, the pace and scale of the outbreak revealed considerable preparedness gaps.

The presence of *A. albopictus* throughout southern China, including Hong Kong, underscores the risk of cross-border transmission. With high volumes of travel between the Greater Bay Area cities, including Shenzhen, Foshan, and Hong Kong, the likelihood of imported infections remains high. In August 2025, Hong Kong confirmed its first imported case of chikungunya in a 12-year-old boy returning from Shunde. While initial cases were successfully contained, the subsequent confirmation of the first locally acquired case in late October 2025 tragically validates this paper's central argument, that is, is the window for pre-emptive containment is exceedingly limited, necessitating an urgent strengthening of public health measures. The case also raises broad concerns about the under-recognition and underreporting of arboviral infections in regions without active surveillance systems. With DENV and ZKV also circulating in overlapping areas, coinfections and misdiagnoses are possible, further complicating clinical management, and epidemiological tracking may be required.

## Vaccination limitations and vector control gaps

Despite the growing global awareness of chikungunya, prevention tools remain limited. Until recently, no licensed vaccine was available. In 2023, the United States approved IXCHIQ^^®^^, a live-attenuated single-dose chikungunya vaccine developed by Valneva (28). The vaccine received conditional approval in the European Union in 2024. However, adoption in Asia has been slow because of regulatory delays and concerns about the vaccine's safety profile in older adult populations (29). Clinical trials are ongoing in several countries, but no large-scale deployment has occurred in mainland China or Hong Kong as of October 2025.

The lack of vaccination coverage leaves vector control as the primary preventive strategy. However, traditional vector control efforts face several challenges (30). Urban environments provide abundant breeding sites for *Aedes* mosquitoes in the form of flower pots, rooftop tanks, drains, and construction sites. The widespread use of insecticides has led to resistance in some mosquito populations, reducing efficacy. Moreover, public engagement in mosquito control tends to decline outside of active outbreak periods, leading to complacency.

Guangdong's 2025 outbreak saw innovative responses, such as the deployment of *Toxorhynchites splendens* (cannibal mosquitoes), to reduce larval density. While such ecological approaches are promising, they require careful monitoring and integration into broad integrated vector management strategies. Public health messaging must also be tailored to local cultures and languages to ensure community buy-in.

## Public health readiness and prevention opportunities

The 2025 chikungunya outbreak offers several lessons for public health authorities in Asia, especially those in cities with high travel volumes and favorable climates for mosquito breeding.

First, cross-border surveillance must be strengthened. The seamless movement of people across the Guangdong, Hong Kong, and Macau borders necessitates real-time data sharing on vector activity and arboviral case detection. A regional early warning system could enhance rapid response to emerging threats (31).

Second, public education campaigns need to be sustained year-round, not just during outbreaks. Raising awareness about the risks of arboviral diseases and the importance of eliminating mosquito breeding sites is essential. This approach includes engaging schools, workplaces, and community organizations in routine cleanup and prevention efforts (32).

Third, travel health advisories should be updated to include chikungunya, especially for outbound travelers to affected areas (33). Returnees with febrile illness should be screened for CHIKV alongside DENV and ZKV, and clinicians should be trained to recognize overlapping symptomatology.

Fourth, vaccination readiness must be prioritized. While the large-scale rollout of the chikungunya vaccine is not yet feasible in Asia, regulatory planning, cold chain logistics, and public communication strategies should be prepared in advance (33). Populations at highest risk, such as the older adult and those in outbreak-prone districts, should be considered in early vaccine access plans once the chikungunya vaccine becomes available locally.

Fifth, community vector control strategies should adopt a whole-of-society approach. Local governments, housing authorities, and district services and community care teams should collaborate on sustained mosquito reduction programs. Digital reporting tools, such as mobile apps to report standing water or mosquito hotspots, can empower communities and support targeted intervention.

Sixth, personal protection must be reinforced as a fundamental pillar of prevention. Given that *Aedes* mosquitoes are active during the day, continuous vigilance is necessary. Personal protection involves educating the public on three key practices: the use of EPA-registered insect repellents containing active ingredients, such as *N*,*N*-diethyl-*meta*-toluamide (commonly known as DEET), picaridin, or IR3535; wearing loose-fitting, light-colored clothing that covers exposed skin; and eliminating all sources of standing water around homes and workplaces (source reduction). Clinicians and travel health advisors must clearly communicate the proper and safe application of the above repellents, including providing guidelines for children and pregnant women (34).

## Conclusion

Chikungunya fever, once considered as a sporadic threat, has rapidly evolved into a regional public health crisis. The unprecedented escalation of the outbreak in Mainland China, now surpassing 17,000 confirmed cases in Guangdong Province, coupled with the definitive confirmation of Hong Kong's first locally acquired case in early November 2025, provides urgent evidence that the window for pre-emptive containment has closed. The emergence of CHIKV in proximity to international hubs underscores the immediate necessity for regional health bodies to abandon a reactive stance. The authors reiterate their call for the immediate adoption of aggressive, cross-jurisdictional proactive strategies rooted in evidence-based public health. These strategies must include prioritizing accelerated vaccine rollouts for high-risk groups; implementing innovative large-scale vector control methods; and establishing robust, standardized cross-border surveillance systems to track and manage this re-emerging threat. The current absence of widespread vaccine coverage in Asia must not delay action. Existing tools, including mosquito control, public education, and travel health guidance, must be leveraged immediately.

Hong Kong, by utilizing its strong health infrastructure and central position within the Greater Bay Area, is uniquely positioned to pilot urgent, integrated chikungunya mitigation and response programs. These efforts should serve as a critical model for other Asian cities now facing an established ecological risk. The time to act is no longer simply limited but critically urgent. Failure to invest and coordinate now will allow chikungunya to become a recurring, costly seasonal health burden permanently embedded within the region's densely populated urban ecosystems in the years to come.
